# Error-Related Brain Activity in Patients With Obsessive-Compulsive Disorder and Unaffected First-Degree Relatives: Evidence for Protective Patterns

**DOI:** 10.1016/j.bpsgos.2021.07.001

**Published:** 2021-07-15

**Authors:** Rosa Grützmann, Christian Kaufmann, Olga A. Wudarczyk, Luisa Balzus, Julia Klawohn, Anja Riesel, Katharina Bey, Michael Wagner, Stephan Heinzel, Norbert Kathmann

**Affiliations:** a Department of Psychology, Humboldt-Universität zu Berlin, Berlin, Germany; b Department of Psychology, Freie Universität Berlin, Berlin, Germany; c Department of Psychology, Universität Hamburg, Hamburg, Germany; d Department of Psychiatry and Psychotherapy, Universitätsklinikum Bonn, Bonn, Germany

**Keywords:** Endophenotype, Error monitoring, Family studies, fMRI, Obsessive-compulsive disorder, Resilience

## Abstract

**Background:**

Indicators of increased error monitoring are associated with obsessive-compulsive disorder (OCD), as shown in electroencephalography and functional magnetic resonance imaging studies. As most studies used strictly controlled samples (excluding comorbidity and medication), it remains open whether these findings extend to naturalistic settings. Thus, we assessed error-related brain activity in a large, naturalistic OCD sample. We also explored which activity patterns might qualify as vulnerability endophenotypes or protective factors for the disorder. To this aim, a sample of unaffected first-degree relatives of patients with OCD was also included.

**Methods:**

Participants (84 patients with OCD, 99 healthy control participants, and 37 unaffected first-degree relatives of patients with OCD) completed a flanker task while blood oxygen level–dependent responses were measured with functional magnetic resonance imaging. Aberrant error-related brain activity in patients and relatives was identified.

**Results:**

Patients with OCD showed increased error-related activity in the supplementary motor area and within the default mode network, specifically in the precuneus and postcentral gyrus. Unaffected first-degree relatives showed increased error-related activity in the bilateral inferior frontal gyrus.

**Conclusions:**

Increased supplementary motor area and default mode network activity in patients with OCD replicates previous studies and might indicate excessive error signals and increased self-referential error processing. Increased activity of the inferior frontal gyrus in relatives may reflect increased inhibition. Impaired response inhibition in OCD has been demonstrated in several studies and might contribute to impairments in suppressing compulsive actions. Thus, increased inferior frontal gyrus activity in the unaffected relatives of patients with OCD may have contributed to protection from symptom development.

Alterations in error processing constitute a robust finding in obsessive-compulsive disorder (OCD) research (1, 2, 3). Pitman (4) proposed that patients with OCD experience excessive error signals that persist despite attempts at behavioral correction (i.e., compulsions) and create uncomfortable “not-just-right” experiences. In line with this model, electroencephalography (EEG) studies found increased amplitudes of the error-related negativity (ERN), an event-related potential that occurs 0 to 50 ms after error commission in response choice tasks (5,6), in patients with OCD (7, 8, 9, 10, 11).

Increased error processing in OCD has also been detected in neuroimaging studies. Greater blood oxygen level–dependent (BOLD) responses to errors in OCD have been observed in the cingulate cortex (12, 13, 14, 15), with local maxima in the midcingulate cortex (MCC) (12,14)^1^ and subgenual anterior cingulate cortex (16, 17, 18). Error-related hyperactivation was also found for regions outside the cingulum, i.e., the anterior insula/frontal operculum and ventromedial prefrontal cortex (17,18). Additionally, reduced activation in the dorsolateral prefrontal cortex (16) was observed. A recent meta-analysis confirmed increased activity in the cingulo-opercular network on error trials in patients with OCD, specifically the bilateral MCC/supplementary motor area (SMA) as well as the right anterior insula/frontal operculum and the anterior lateral prefrontal cortex (19). Although multiple studies demonstrated excessive activity of error-processing networks in OCD, they mostly investigated highly selective samples that are relatively homogeneous in clinical characteristics such as comorbidity, medication, and symptom dimensions. Thus, generalizability to OCD populations in routine therapeutical care may be limited. Therefore, we investigated error-related brain activity in a large, naturalistic sample of 84 patients with OCD. We hypothesized that patients with OCD should exhibit increased activation of regions within the cingulo-opercular network on error trials.

EEG studies have yielded evidence that altered error monitoring might reflect a vulnerability endophenotype for OCD. Specifically, increased ERN amplitudes are also observed in unaffected first-degree relatives (FDRs) of patients with OCD (8,20,21). Furthermore, increased ERN in OCD is independent of symptom expression (22) and persists despite successful treatment (7,23). Endophenotypes are quantitative biological or cognitive markers that are less complex and therefore may be more closely related to the genetic underpinnings than the clinical syndrome (24). Although twin and family studies provide evidence for small to moderate genetic effects in OCD (25), OCD appears to be influenced by multiple genetic and environmental factors, and replicable evidence on specific genetic alterations has not yet emerged. Thus, identifying vulnerability endophenotypes of OCD is thought to further extend knowledge about the condition’s etiology (26). Additionally, endophenotypes may help to identify and specifically target individuals at risk for developing a disorder (27,28).

It has not been investigated yet whether altered activation of regions within the error monitoring network measured with functional magnetic resonance imaging (fMRI) qualifies as a vulnerability endophenotype. Thus, in the present study, error-related BOLD responses were also assessed in a sample of unaffected FDRs of patients with OCD. A similar activity pattern in patients with OCD and FDRs as opposed to healthy control (HC) subjects would yield evidence for an endophenotype candidate. As EEG studies report increased ERN amplitudes in unaffected FDRs of patients with OCD (8,20,21) and our previous study identified the MCC and SMA as generators of the ERN in patients with OCD (29), we expected endophenotypic error-related hyperactivation patterns to be localized in these regions.

Investigating unaffected FDRs can also provide insight into protective factors. FDRs share genetic and environmental risk factors with patients with OCD. Still, they did not develop the disorder phenotype. To clarify which group difference patterns might be expected, it is important to distinguish between assets and protective factors. Assets are variables that influence development in a positive way, regardless of whether a risk factor is present, and should be associated with positive outcomes across the whole population (30,31). In contrast, a protective factor influences outcome only in the presence of risk factors by moderating their effect. As FDRs are specifically chosen based on the presence of a risk factor (i.e., family history of OCD) and still did not develop the disorder phenotype yet, they are more likely to have benefitted from the presence of a protective factor than HC participants (31). Hence, activity patterns that are specific to unaffected relatives as compared with patients with OCD and HC participants might represent protective factors.

## Methods and Materials

### Participants

Initially, 98 patients with OCD, 46 unaffected FDRs of patients with OCD but without an individual history of diagnosed OCD, and 117 HC participants without a history of OCD and with no current diagnosis of any psychiatric disorder took part in the study. Post hoc exclusion owing to insufficient data quality (described in detail in Data Analysis) resulted in a final analysis sample of 84 patients with OCD, 99 HC participants, and 37 FDRs.

All participants received verbal and written explanation of the purpose and procedures of the study, gave their written informed consent in accordance to the ethical guidelines of the Declaration of Helsinki, and received 10€ per hour for their participation. The study was approved by the ethical review board of the Humboldt-Universität zu Berlin. Participants were between 18 and 65 years of age, had normal or corrected-to-normal vision, and reported no history of head trauma or neurological diseases.

Patients were diagnosed by trained clinicians using the German version of the Structured Clinical Interview for DSM-IV (32). All patients fulfilled DSM-IV criteria for OCD and were on a waitlist for cognitive behavioral treatment at the OCD outpatient clinic of the Humboldt-Universität zu Berlin. Thirty-five patients reported taking one or more psychotropic medications in the last 3 months (selective serotonin reuptake inhibitor, *n* = 28; selective serotonin-norepinephrine reuptake inhibitor, *n* = 4; tricyclic antidepressant, *n* = 6; tetracyclic antidepressant, *n* = 1; atypical antipsychotic, *n* = 1; other medication, *n* = 2). Fifty-nine patients had current comorbid diagnoses including affective disorder (*n* = 50), anxiety disorder (*n* = 8), and somatoform disorder (*n* = 1). Patients with comorbid psychotic or substance abuse disorders were excluded.

FDRs were recruited via patients with OCD, who gave written informed consent for contacting their relatives. FDRs were included only if they reported no past or present history of OCD. Additional exclusion criteria applied to unaffected FDRs of patients with OCD were lifetime diagnosis of psychotic, bipolar, or substance abuse disorder, and psychoactive medication in the past 4 weeks. As evident in the Supplement, FDRs and HC participants did not differ in depression and trait anxiety measures. OCD symptoms were slightly higher in FDRs than in HC participants but were significantly lower than in patients with OCD and well below the clinical cutoff.

The HC group was matched for gender, age, education, and handedness to the patients with OCD. Exclusion criteria for the control group were psychoactive medication in the past 3 months, any current or past Axis I disorder, and family history of OCD in FDRs.

In patients, obsessive-compulsive symptoms were assessed with the Yale-Brown Obsessive Compulsive Scale (clinician rating) (33) and depressive symptoms with the Montgomery–Åsberg Depression Rating Scale (34) by trained clinicians. All participants additionally completed the Obsessive Compulsive Inventory–Revised (35), Beck Depression Inventory-II (36), and State–Trait Anxiety Inventory (37). Verbal intelligence was measured with a German vocabulary test (Wortschatztest) (38).

### Stimuli and Procedures

**Figure 1:**
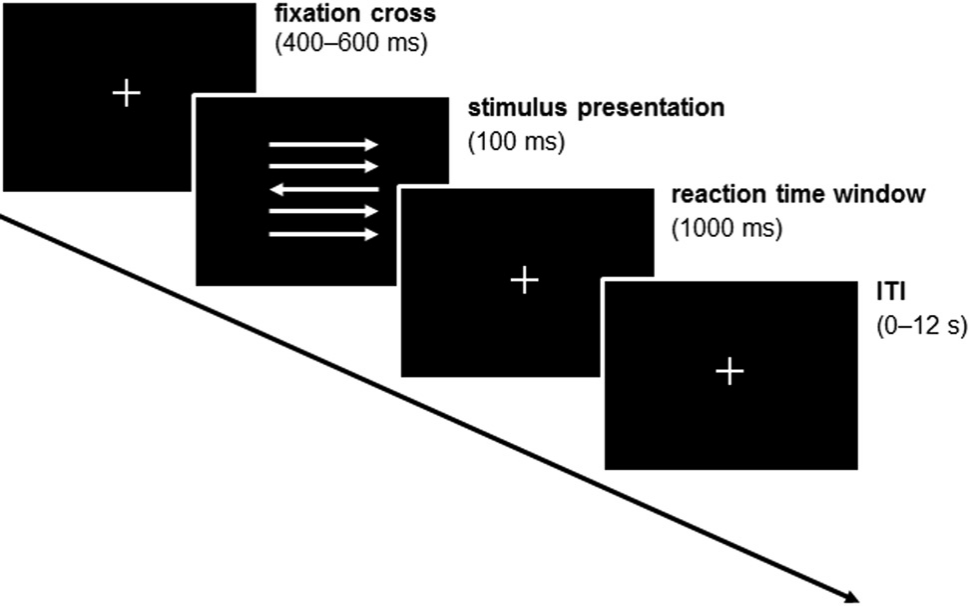
Experimental design of the flanker task. Participants were instructed to respond fast and accurately with their left or right thumb to the direction of the central target arrow. ITI, intertrial interval.

An arrow version of the flanker interference task (Figure 1) (29,39) was administered using Presentation Software (version 18.1; Neurobehavioral Systems; https://www.neurobs.com/menu_presentation/menu_download/current). Visual stimuli were projected by means of a mirror system attached to the head coil (viewing distance approximately 72 cm) and response times were recorded.

Participants were instructed to respond as quickly and accurately as possible to the direction of a centrally located horizontal target arrow. In half of the trials the target was flanked by arrows pointing in the same direction (congruent trials), and in the other half the target was flanked by arrows pointing in the opposite direction (incongruent trials). Across trials, the direction of the target was varied pseudo-randomly. Each trial started with a fixation cross that was presented at the center of the screen for 400 to 600 ms. Then, the arrows were projected for 100 ms, followed by a response time window (maximal 1000 ms). The trial duration varied between 1500 and 1700 ms. A jittered intertrial interval between 0 and 12 seconds preceded the next trial in order to ensure optimized estimation of the BOLD response.

The task consisted of 480 trials (240 incongruent) presented in six blocks, resulting in a total duration of approximately 24 minutes. Participants completed 20 practice trials outside the scanner.

### fMRI Data Acquisition

Imaging data were acquired at the Berlin Center for Advanced Neuroimaging with a 3T Siemens Trio MR system (Siemens Corp.) equipped with a 32-channel head coil. Prior to acquisition of functional images, 192 anatomical slices were acquired using a T1-weighted sagittal sequence (2440-ms repetition time, 4.81-ms echo time, 8° flip angle, 234-mm field of view, 256 × 256 matrix size, isotropic 0.91-mm spatial resolution). Additionally, a T2-weighted sagittal sequence with 192 anatomical slices was measured (5000-ms repetition time, 499-ms echo time, 120° flip angle, 256 × 258 matrix size, isotropic 0.91-mm spatial resolution). During task performance, 730 functional whole-brain volumes were acquired using a T2∗-weighted single-shot echo-planar gradient imaging sequence (1940-ms repetition time, 30-ms echo time, 78° flip angle, 192-mm field of view, 64 × 64 matrix size, 3 × 3 × 3.75 mm voxel size). Thirty-two axial slices with a thickness of 3 mm (gap = 0.75) were acquired in a descending order aligned parallel to the anteroposterior commissure line. To reduce head motion, the subject’s head was immobilized by a vacuum head cushion. Earplugs were used to attenuate background noise.

### fMRI Data Preprocessing

Preprocessing and analysis of fMRI data were performed using SPM12 (Statistical Parametric Mapping Version 7487; http://www.fil.ion.ucl.ac.uk/spm). After conversion from DICOM to NIfTI file format, the images were manually reoriented along the anteroposterior commissure line. The first four volumes of each functional run were discarded to allow for T1 saturation effects to stabilize. To correct for interscan head movements, all images were realigned to the average volume of all images using a least-square approach and a six-parameter rigid-body spatial transformation with a second-degree B-spline interpolation. Functional images were slice-time corrected using the acquisition time of the middle slice as reference. Each subject’s structural scan was coregistered with the mean realigned functional image. T1-weighted images were segmented into gray matter, white matter, and cerebrospinal fluid (by integrating information of the T2-weighted image). The segmented gray matter was then spatially normalized to the standard template provided by the Montreal Neurological Institute by applying a 12-parameter affine transformation, followed by a nonlinear warping using basis functions (40). The resulting normalization parameters were applied to the functional images, and all volumes were resampled to a 2-mm isotropic resolution and spatially smoothed with an 8-mm full width at half maximum kernel.

### Data Analysis

Eight participants showed excessive head movement with more than 3-mm estimated translational or 2° rotational movements (i.e., more than voxel size; 3 patients with OCD, 2 FDRs, 3 HC participants). Five participants were excluded due to misses in more than 20% of trials (1 patient with OCD, 1 FDR, 3 HC participants), and 2 participants were excluded due to error commission on more than 30% of the trials (2 HC participants). As we aimed to analyze error-related brain activity, 26 participants who committed errors on <5% of the incompatible trials were excluded (10 patients with OCD, 6 FDRs, 10 HC participants).

#### BOLD Activity Analyses

To analyze functional brain data, a general linear model was specified for each subject. Two regressors of interest were defined: one for error (by modeling incongruent error > incongruent correct) and one for conflict (by modeling incongruent correct > congruent correct). Errors predominantly occur in incompatible trials. Thus, error-related brain activity comprises conflict processing and error processing. Therefore, the conflict contrast was defined as the comparison condition, in order to identify activity that is specific to error processing, while controlling for conflict effects. The regressors were convolved with a canonical hemodynamic response function. Six motion parameter vectors (three translational and three rotational parameters) were modeled as regressors of no interest to account for variance related to head movement.

The contrast images obtained from the first-level analysis (error, conflict) were entered into a second-level analysis. Group-level data were analyzed using GLM Flex (Version Fast4; http://mrtools.mgh.harvard.edu/index.php/Downloads), a second-level analysis tool that allows for specification of partitioned error terms for within-group and between-group comparisons. A repeated-measures analysis of variance including response type (error, conflict) as within-subjects factor and group (OCD, HC, FDR) as between-subjects factor was defined. As FDRs were significantly older than HC participants and patients with OCD (see Supplement), age was included as a covariate. Additionally, correlations between age and BOLD activity were computed for clusters showing altered activity in FDRs (see Supplement). To correct for multiple comparisons, we applied an extent threshold correction as defined by Monte Carlo simulations (3DClustSim; implemented in AFNI [National Institutes of Health, Bethesda, MD] (41). For a threshold at the voxel level of *p* < .001 uncorrected, and with spatial properties of the current study, 10,000 simulations resulted in an extent threshold of 56 voxels at *p* < .05. Beta values from significantly activated clusters (defined as 5-mm-sphere radius around the Montreal Neurological Institute coordinates of peak voxel) in the whole brain interaction were extracted and submitted to a response-type-by-group repeated-measurement analysis of variance in SPSS (Version 27; IBM Corp.) and followed up with post hoc *t* tests aimed at disentangling the interaction. Results were visualized using FIVE (Functional Image Visualization Environment; Version MRtools_2015-08-21; http://mrtools.mgh.harvard.edu/index.php/Downloads) and xjView (Version 9.6; http://www.alivelearn.net/xjview). As part of the patient sample received medication, control analyses comparing medicated and unmedicated patients are reported in the Supplement.

## Results

Results of analyses of clinical, demographical, and behavioral data are presented in the Supplement.

### fMRI Results

Table 1 depicts a summary of neuronal activations for the effect of response type across groups and for the group-by-response-type interaction.

**Table 1:** Peak Activations for Whole-Brain Analyses: Response-Type Effect (Error vs. Conflict) and Response-Type-by-Group Interaction

Peak Activity Neural Region	Hemisphere	x	y	z	z_max_	k
Response-Type Effect (Across Groups)						
Error > conflict						
Inferior frontal gyrus extending to anterior cingulate cortex	R	44	24	−8	15.23	14160
Inferior frontal gyrus extending to insula	L	−36	22	−8	14.64	4888
Supramarginal gyrus	R	56	−44	34	14.67	4757
Supramarginal gyrus	L	−58	−50	32	12.54	2579
Midcingulate cortex	L, R	−2	−20	32	6.96	428
Precuneus	R	8	−70	42	4.63	148
Middle temporal gyrus	L	−52	−28	−8	4.88	126
Inferior temporal gyrus	R	50	−6	−34	7.86	96
Cerebellum	L	−24	−68	−32	9.68	1049
Conflict > error						
Middle occipital gyrus	L, R	30	−84	2	11.79	35976
Middle frontal gyrus	L	−20	28	46	8.44	725
	R	22	30	42	5.50	165
Inferior frontal gyrus	L	−22	34	−10	7.07	179
	R	26	36	−8	6.49	83
Cerebellum	R	42	−68	−38	6.42	102
Group-by-Response-Type Interaction						
Inferior frontal gyrus (pars orbitalis)	R	44	26	−12	9.94	93
Inferior frontal gyrus (pars orbitalis) extending to insula	L	−32	22	−12	13.36	125
SMA extending to preSMA	R, L	2	−16	64	10.01	120
Precuneus	R	12	−46	62	9.94	165
Postcentral gyrus	L	−24	−44	66	9.45	80

L, left; R, right; SMA, supplementary motor area.

#### Response-Type Effect Across Groups

Across groups, participants showed stronger activation to errors as compared with conflict trials in nine clusters encompassing the right inferior frontal gyrus (IFG) extending to the anterior cingulate, the left IFG extending to the insula, the bilateral supramarginal gyrus, the bilateral MCC, the right precuneus, the left middle temporal gyrus, the right inferior temporal gyrus, and the cerebellum.

In the reversed contrast, i.e., conflict > errors, participants showed increased activation in six clusters within the middle occipital gyrus, bilateral IFG (pars orbitalis), bilateral middle frontal gyrus, and cerebellum.

#### Group-by-Response-Type Interaction

**Figure 2:**
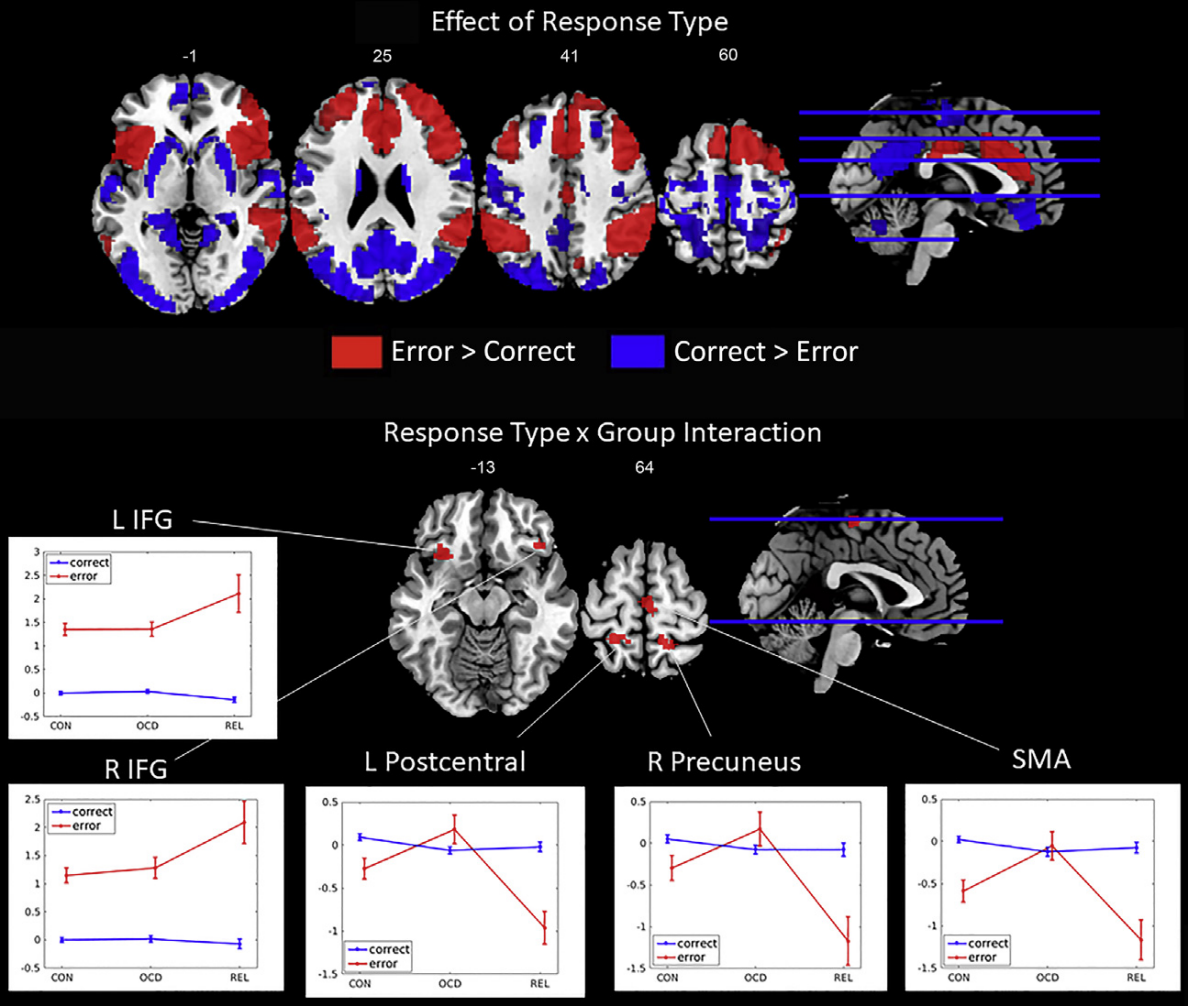
**(A)** Brain activity in response to errors vs. conflict trials. Red indicates regions showing increased activation to errors as compared with conflict trials, and blue indicates decreased activation to errors as compared with conflict trials. **(B)** Brain activity in the whole-brain group-by-response-type interaction as well as a depiction of beta values for significantly activated clusters (cluster-corrected *p* < .05, k = 56). IFG, inferior frontal gyrus; L, left; R, right; SMA, supplementary motor area. CON, healthy control participants; OCD, obsessive-compulsive disorder; REL, first-degree relatives.

The group-by-response–type interaction revealed five clusters within the left IFG (pars orbitalis) extending to the insula, the right IFG (pars orbitalis), the right precuneus, the left postcentral gyrus, and the SMA extending to the preSMA (see Figure 2 and Table 1).

Post hoc tests showed that the error-related activation in the SMA was stronger in patients with OCD (mean = −0.05, SD = 1.54) as compared with HC participants (mean = −0.59, SD = 1.30; *t*_181_ = 2.54, *p* = .012) and FDRs (mean = −1.16, SD = 1.44; *t*_119_ = 3.73, *p* < .001). Patients with OCD also showed stronger error-related activation in the left postcentral gyrus (mean = 0.18, SD = 1.52) than HC participants (mean = −0.27, SD = 1.20; *t*_181_ = 2.26, *p* = .025) and FDRs (mean = −0.96, SD = 1.15; *t*_119_ = 4.07, *p* < .001). Error-related activation of the right precuneus was also stronger in patients with OCD (mean = 0.17, SD = 1.87) than in HC participants (mean = −0.30, SD = 1.48; *t*_181_ = 1.89, *p* = .061) and in FDRs (mean = −1.17, SD = 1.77; *t*_119_ = 3.70, *p* < .001). FDRs showed significant deactivation in these regions compared with HC participants (SMA [*t*_134_ = 2.24, *p* = .027], left postcentral gyrus [*t*_134_ = 3.00, *p* = .003], right precuneus [*t*_134_ = 2.91, *p* = .004]).

Error-related activity of the right IFG was significantly increased in FDRs (mean = 2.09, SD = 2.30) as compared with patients with OCD (mean = 1.28, SD = 2.09; *t*_119_ = 2.16, *p* = .033) and HC participants (mean = 1.15, SD = 1.31; *t*_45.10_ = 2.99, *p* = .023). Error-related activity of the left IFG also tended to be stronger in FDRs (mean = 2.11, SD = 2.41) as compared with patients with OCD (mean = 1.36, SD = 1.38; *t*_46.70_ = 1.78, *p* = .081) and HC participants (mean = 1.35, SD = 1.29; *t*_43.95_ = 1.83, *p* = .075).

## Discussion

The present study pursued two goals. The first one was to replicate previous findings of error-related hyperactivation in the cingulo-opercular network in a naturalistic sample of patients with OCD. The second goal was to identify patterns of error-related neuronal activation in unaffected FDRs of patients with OCD that might qualify as vulnerability endophenotypes or protective factors.

Analysis of fMRI data across the whole sample confirmed typical activity of the error monitoring network, including the MCC, bilateral inferior parietal cortices, and bilateral anterior insula/frontal operculum (42,43). In line with previous studies (19), the group comparison revealed increased error-related activity of regions in the cingulo-opercular network in patients with OCD as compared with HC participants and FDRs. Specifically, patients with OCD showed stronger activation in the bilateral SMA. Additional error-related hyperactivation was observed in the right precuneus and left postcentral gyrus. Thus, the present analysis demonstrates that error-related hyperactivation in OCD is also observed in a more representative sample exhibiting variability in clinical characteristics such as comorbidity, medication, and symptom dimension.

Confirming previous results (29), group differences were located in the SMA, rather than in the MCC, which has been identified as the main generator of the ERN in healthy populations (44, 45, 46). Previous research indicated that the MCC may be mainly involved in recruiting reactive cognitive control, while conversely the SMA is involved in recruiting proactive cognitive control (47,48). Thus, performance monitoring alterations in OCD might be characterized by inflexible and excessive control recruitment, which is also supported by behavioral data implying reduced strategic control adaptation (49,50) and by principal component analysis of EEG data (51).

Additional error-related hyperactivation was observed in the right precuneus and the left postcentral gyrus. Both regions contribute to the default mode network (DMN), which comprises brain regions that are deactivated during tasks requiring externally oriented attention but activated during passive rest states (52, 53, 54). The DMN, especially its posterior division including the precuneus, is also activated during internally focused attention such as autobiographic memory, self-referential processing, and future thinking (54, 55, 56, 57, 58, 59). Previous studies have demonstrated abnormal intrinsic functional connectivity within the DMN and between the DMN and the cingulo-opercular and frontoparietal networks in OCD (60, 61, 62). These alterations may contribute to the inability of patients with OCD to disengage from internally generated scenarios and thoughts when performing everyday tasks requiring external attention (62). Thus, the increased error-related activity of the precuneus and the postcentral gyrus might reflect increased self-referential or future-oriented error processing in OCD (e.g., worrying about the possible consequences of the error). This is in line with reports of increased harm avoidance and perfectionism in OCD (20,63, 64, 65, 66) and supports the assumption that patients with OCD exhibit increased affective responses to errors (18,29).

In order to identify possible brain activation patterns that might constitute vulnerability endophenotypes or protective factors, unaffected FDRs of patients with OCD were investigated. EEG studies have detected increased ERN amplitudes in FDRs of patients with OCD (8,20,21). As the ERN has major generators in the MCC and SMA (29,44, 45, 46), increased activity of these regions was expected in FDRs. However, we found no evidence for increased activity of these regions in FDRs. Notably, FDRs exhibited increased ERN amplitudes, as supported by a separate analysis of EEG data collected from a largely overlapping sample (20). The current data implicate that error-related EEG and fMRI measurements are less parallel than expected, possibly owing to the differential temporal sensitivity of the two methods. In line with this distinction, increased ERN in OCD is state independent (7,23), thereby fulfilling another criterion for endophenotypes (24), while error-related activity of the MCC/SMA was positively correlated with symptom severity in several studies (12,67). At present, the current results argue against error-related BOLD increase in the MCC/SMA as a robust vulnerability endophenotype of OCD.

Relatives, compared with HC participants and patients with OCD, exhibited increased error-related activation of the bilateral IFG, a region implicated in response inhibition (68,69). Multiple studies have yielded evidence for behavioral response inhibition deficits in OCD (70,71). In line with that, Norman *et al.* (19) detected a decreased BOLD response in the inferior temporal lobe in OCD on response conflict trials in their meta-analysis. This was interpreted as evidence for a general inhibitory deficit in OCD that plays a role in symptom generation. Specifically, impairments in implementing corrective inhibitory control following the detection of goal-incongruent behaviors might result in patients’ becoming stuck in compulsive loops. Insufficient inhibition of intrusive thoughts or related affective responses might also contribute to symptom generation. Both patients with OCD and FDRs report increased harm-avoidance tendencies (63,64,72,73). Thus, both groups appear to show increased performance monitoring and increased (emotional) responses to errors, possibly resulting in behavioral impulses for remedial actions (e.g., checking, washing). While inhibitory deficits may contribute to symptom generation in clinical patients, intact or even increased inhibitory functions in relatives might prevent them from acting on these impulses, thereby exerting their protective function. Supportive of a potential protective role of increased IFG activity, FDRs show stronger error-related deactivation of the DMN compared with patients with OCD, which may indicate less self-referential error processing. As the increased IFG activity in FDRs extends into the insula, it could represent increased but, compared with patients with OCD, more spatially extended hyperactivity of the cingulo-opercular network. However, reduced error-related activity of the SMA in FDRs contradicts this concept. To distinguish between these possible interpretations, increased IFG activity in FDRs should be further explored, i.e., by functional connectivity analyses or by linking it to behavioral indicators of inhibition. A pattern of selectively increased activity in unaffected relatives compared with patients with OCD and HC participants has also been reported for the premotor cortex in the n-back task (74). Identifying protective factors can beneficially complement research on vulnerability. Endophenotype research might help to identify at-risk individuals who can then receive interventions enhancing functions that have previously been identified as protective factors.

Some limitations need to be considered. The present study aimed to extend previous findings to naturalistic populations. Thus, the OCD group was heterogeneous regarding comorbidity, medication, and symptom dimensions. In contrast to recent meta-analyses (1,19), alterations in error-related brain activity were stronger in medicated patients (see Supplement). This further illustrates that hyperactive error monitoring measured by fMRI might be less robust and therefore less suited as an endophenotype than the ERN measured with EEG. As a substantial part of the relatives group consisted of patients’ parents (*n*_parents_ *=* 27, *n*_siblings_ = 8, *n*_children_ = 2), this group was significantly older than the OCD and HC groups. To control for age effects, age was included as a covariate in all analyses (see also Supplement). Finally, the relatives in this sample exhibited slightly higher OCD symptom levels than HC individuals. However, symptoms were well below the clinical range and relatives did not fulfill diagnostic criteria for OCD as confirmed by structured clinical interviews.

In sum, the present study provides evidence for altered error-related brain activity in a naturalistic sample of patients with OCD. Error-related hyperactivity was mainly located in the SMA. Furthermore, error-related deactivation of the DMN was decreased. Taken together, these patterns imply increased affective and self-referential error processing in OCD. Vulnerability endophenotype patterns of increased error-related activity in both patients and relatives, as commonly observed in EEG studies, were not detected. However, relatives exhibited increased error-related activity in the bilateral IFG, pointing to increased inhibition, which might constitute a protective factor that should be studied in more detail.

## Supplementary Material

Supplementary Material

Key Resources Table

## Footnotes

1 Subregions of the cingulate cortex were labeled according to the three-region model by Vogt (75). Based on the alternative two-region model, the MCC is also referred to as dorsal anterior cingulate cortex.
