# Supplementary material for: Error-Related Brain Activity in Patients With Obsessive-Compulsive Disorder and Unaffected First-Degree Relatives: Evidence for Protective Patterns
Source: Biol Psychiatry Glob Open Sci. 2021 Jul 15;2(1):79–87. doi: 10.1016/j.bpsgos.2021.07.001 (PMC9616249; doi:10.1016/j.bpsgos.2021.07.001)
Supplement: Supplementary file 1 — Supplementary Material [file mmc1.pdf]

Error-Related Brain Activity in Obsessive-Compulsive Disorder Patients and Unaffected First-Degree Relatives: Evidence for Protective Patterns

Supplement

Clinical, Demographic, and Behavioral Data Analyses

Clinical, demographic, and behavioral data were analyzed using IBM SPSS Statistics (Version 22.0). Data were compared between groups (OCD, FDR, HC) using one-way analyses of variance (ANOVAs) and chi-square tests. For behavioral analyses, all trials with response times (RTs) deviating more than 2.5 SD from the intra-individual mean of the respective condition were excluded. As errors in congruent trials were rare, they were excluded from all analyses. Post-error slowing was calculated as a difference in RT between post-error and the associated pre-error trial. This method proposed by Dutilh et al. (1) was used to quantify post-error slowing in order to avoid confounding with global fluctuations in motivation and response caution. Repeated measures analysis of covariance (ANCOVA) was used to analyze RT in the task, and included group (OCD, FDR, HC) as between-subject factor and response type (congruent correct, incongruent correct, and incongruent error) as within-subject factor. In case of significant main effect or interaction, post hoc comparisons were executed using *t*-tests. As age differed significantly between groups, it was included as a covariate in the behavioral analyses of error rates, RT, and post-error slowing. In case the covariate age was significant, its relationship with a variable of interest was tested using Spearman's correlation.

Clinical, Demographic, and Behavioral Data Results

Table S1 shows clinical, demographic, and behavioral measures and the statistics for the comparison between groups derived from univariate ANOVAs.

SCID-IV interviews showed that nine FDR fulfilled diagnostic criteria for past or present Axis-I disorders such as past major depressive episode (n = 3), past social phobia (n = 2), current specific phobia (n = 1), past specific phobia (n = 1), current generalized anxiety disorder (n = 1), past panic disorder (n = 1), past anorexia nervosa (n = 1) and present skin picking disorder (n = 4).

While OCD patients and HC did not differ in age, $t(181) = 0.12$, p =. 903, FDR were significantly older than OCD patients, $t(46.83) = 4.91$, $p < .001$, and HCs, $t(48.09) = 4.81$, $p < .001$. The groups did not differ in gender distribution and verbal intelligence. OCD patients scored higher on clinical questionnaires as compared to FDR (OCI-R: $t(115.34) = 11.02$, $p < .001$, BDI-II: $t(118.56) = 10.87$, $p < .001$, STAI Trait: $t(99.89) = 12.36$, $p < .001$) and HC (OCI-R: $t(93.44) = 15.44$, $p < .001$, BDI-II: $t(95.42) = 12.98$, $p < .001$, STAI Trait: $t(135.18) = 14.70$, $p < .001$). FDR and HC did not differ in depression and trait anxiety measures. However, FDR reported slightly higher OCD symptoms than healthy participants (OCI-R: $t(43.37) = 2.26$, $p = .029$).

Univariate ANCOVAs revealed that OCD patients, FDR, and HCs did not significantly differ in error rates or post-error slowing. Repeated measures ANCOVA on RTs to incongruent correct, incongruent error, and congruent correct trials revealed a significant main effect of response type, $F(2, 432) = 23.96$, $p < .001$, indicating faster responses to congruent (M = 420.80, SD = 50.36) than to incongruent correct trials (M = 477.53, SD = 49.08), $t(220) = 51.11$, $p < .001$, and to incongruent error trials (M = 429.00, SD = 92.00) as compared to incongruent correct trials, $t(220) = 11.41$, $p < .001$. A main effect of group $F(2, 216) = 3.09$, p =.048, and its interaction with response type, $F(4, 432) = 7.95$, $p < .001$, indicated slower responses in FDR to incongruent correct trials as compared to OCD, $t(119) = 2.15$, p =.033. A significant main effect of the covariate age, $F(1, 216) = 36.28$, $p < .001$, indicated that increased age was linked to increased RTs, $r = .364$, $p < .001$. The interaction of the covariate age with

response type, $F(2, 432) = 5.02$, $p = .007$, additionally specified that this effect was most pronounced for incongruent correct trials, $r = .435$, $p < .001$, but also present for congruent correct trials, $r = .355$, $p < .001$, and incongruent error trials, $r = .277$, $p < .001$. Response time data is presented in Figure S2.

	**HC**	**OCD**	**FDR**
**Demographic Data**			
N	99	84	37
Age (years)	31.4 (9.7)	31.6 (9.5)	44.9 (15.6)
Gender (M:F)	41:58	39:45	13:24
Verbal IQ (WST)	104.6 (10.0)	103.5 (10.0)	107.1 (9.5)
**Clinical Data**			
Y-BOCS	-	22.2 (5.0)	-
MADRS	-	13.7 (9.8)	-
BDI II	2.4 (3.4)	19.3 (11.5)	3.3 (4.7)
OCI-R	3.7 (3.4)	25.9 (12.8)	6.3 (6.7)
STAI trait	32.5 (7.2)	53.6 (11.1)	32.1 (7.5)
**Behavioral Data**			
% Errors	6.1 (6.8)	5.7 (6.0)	4.8 (3.2)
RT: Incongruent Correct	477 (49)	471 (50)	492 (46)
RT: Incongruent Error	432 (84)	433 (110)	409 (57)
RT: Congruent Correct	421 (51)	417 (53)	428 (44)
Post-Error Slowing	42 (34)	40 (33)	62 (38)

Table S1. Demographic, clinical, and behavioral data of patients with OCD, unaffected first-degree relatives (FDR), and healthy participants (HC). For metric data, mean and standard deviation (in parentheses) are provided. Response times and post-error slowing are reported in milliseconds.

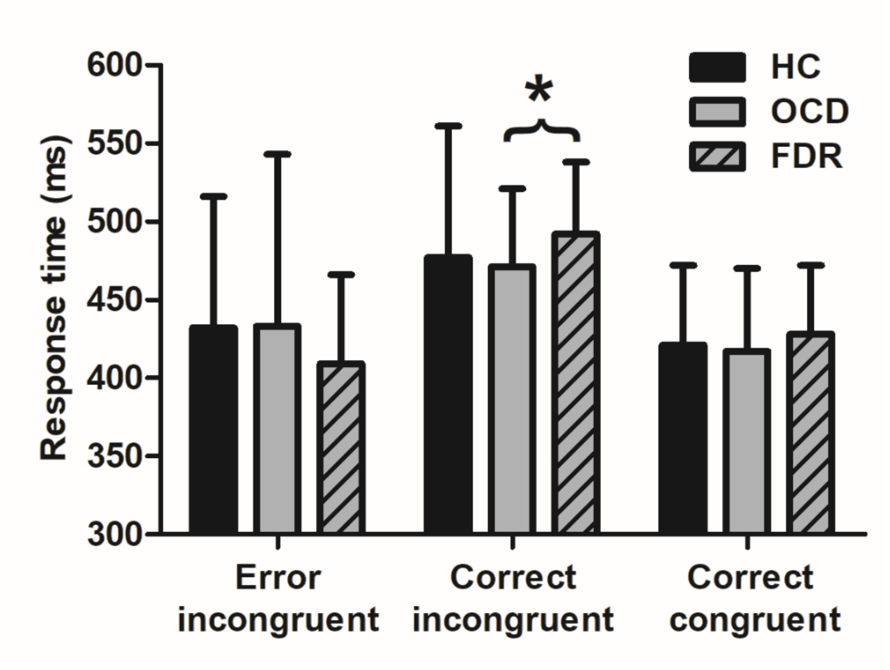

*Figure S1. Response time in milliseconds ± standard deviation by response type (incongruent error, incongruent correct, and congruent correct) and group (healthy controls [HC], patients with OCD [OCD], and relatives [FDR]). * indicates significant comparisons (p < .05)*

Correlations with age

As FDR were significantly older than HC and OCD, age differences may have contributed to the between-group differences in error-related brain activity. To explore possible confounding association patterns, correlations were computed between age and error-related activation of the brain regions exhibiting significant group differences in the ANOVA (quantified as mean beta values from a 5 mm sphere radius around the MNI coordinates of peak voxels of the respective clusters). Correlations were computed across the whole group and separately for each group.

	All		OCD		HC		FDR	
	r	*p*	*r*	*p*	*r*	*p*	*r*	*p*
Left IFG	-.054	.427	.089	.422	-.099	.331	**-.376**	**.022**
Right IFG	-.015	.829	.002	.989	-.058	.568	**-.391**	**.017**
SMA	**-.131**	**.053**	-.140	.204	-.142	.160	.226	.179
Precuneus	**-.134**	**.047**	-.095	.391	**-.202**	**.045**	.265	.113
PCG	-.113	.094	-.079	.477	-.056	.584	.180	.286

Table S2. Correlations between age and error-related activity of the brain regions showing between-group differences in the ANOVA in the whole sample and within each group. Significant correlations (p < .05) are marked in bold font.

Age was negatively correlated with the error-related activation of the left and right IFG in FDR, indicating that higher age was associated with reduced error-related activity of the IFG. The direction of the correlation is opposite to the increased activation of the IFG in FDR that was observed in the main analysis. Thus, these correlation patterns do not support the notion that the IFG effect in FDR is mainly driven by the older age of this group. On the contrary, data indicates that differences in IFG activation may even be underestimated in the current sample as they are less expressed in older FDR. None of the other correlations in FDR reached significance.

Across the whole group and in HC, error-related activation of the precuneus was negatively correlated with age, indicating stronger deactivation of the DMN in younger participants as previously

shown (2). Furthermore, error-related activation of the SMA was negatively correlated with age. This is in line with decreased ERN amplitudes in older participants (3, 4). However, note that both correlations are numerically small and significance may be driven by the large sample (n = 220).

Comparison between medicated and unmedicated OCD patients

Thirty-five of the OCD patients received psychoactive medication at the time of study. The following tables illustrates demographic and clinical characteristics and behavioral data in unmedicated and medicated patients.

	OCD unmedicated		OCD medicated			
	n = 49		*n* = 35			
	M	SD	M	SD	*t*	*p*
Age	31.53	9.50	31.74	9.56	-0.10	.920
WST IQ	102.46	10.63	104.97	8.86	-1.14	.258
OCI-R	24.69	11.73	27.46	14.09	-0.98	.331
Y-BOCS	21.88	5.21	22.57	4.74	-0.62	.534
BDI II	18.69	10.52	20.03	12.81	-0.52	.602
MADRS	13.24	9.57	14.29	10.17	-0.48	.633
Age of Onset	24.15	10.79	21.24	8.92	1.31	.195
Illness duration	7.61	8.52	10.27	9.78	-1.26	.213
Correct RT incom (ms)	472.51	47.27	469.92	53.76	0.23	.816
Correct RT com (ms)	417.55	50.41	416.41	56.73	0.10	.923
Error RT (ms)	438.14	113.20	426.70	106.42	0.47	.641
Error rate (%)	9.88	7.68	9.68	8.09	0.11	.909
PES (ms)	37.60	31.34	43.54	35.55	-0.81	.421

Table S3. Demographic and clinical characteristics and behavioral data in unmedicated and medicated patients.

In order to assess whether the effects detected in the whole brain analyses differ between medicated and unmedicated OCD patients, the post-hoc test were repeated with four groups (OCD medicated, OCD unmedicated, FDR, HC). These analyses showed that in the present sample alterations were more expressed in medicated than in unmedicated patients.

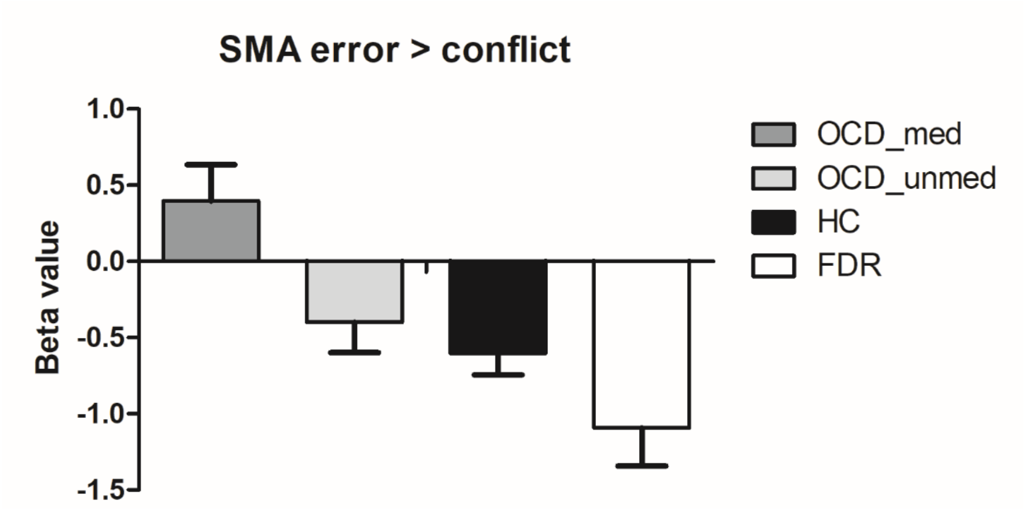

Figure S2. Error-related activity of the supplementary motor area (SMA) ± standard error in medicated OCD patients (OCD_med), unmedicated OCD patients (OCD_unmed), healthy control participants (HC) and unaffected first-degree relatives of OCD patients (FDR).

Error-related activation of the SMA was stronger in medicated patients than in unmedicated patients, $t(82) = 2.39$, $p = .019$. Consequently, medicated patients showed stronger error-related SMA activation than HC, $t(132) = 3.55$, $p = .001$, while the difference did not reach significance in unmedicated OCD patients, $t(146) = 0.90$, $p = .368$. FDR showed decreased activation of the SMA compared to both patient groups, all t > -2.65, all $p < .010$, and to HC, $t(134) = -2.24$, $p = .027$.

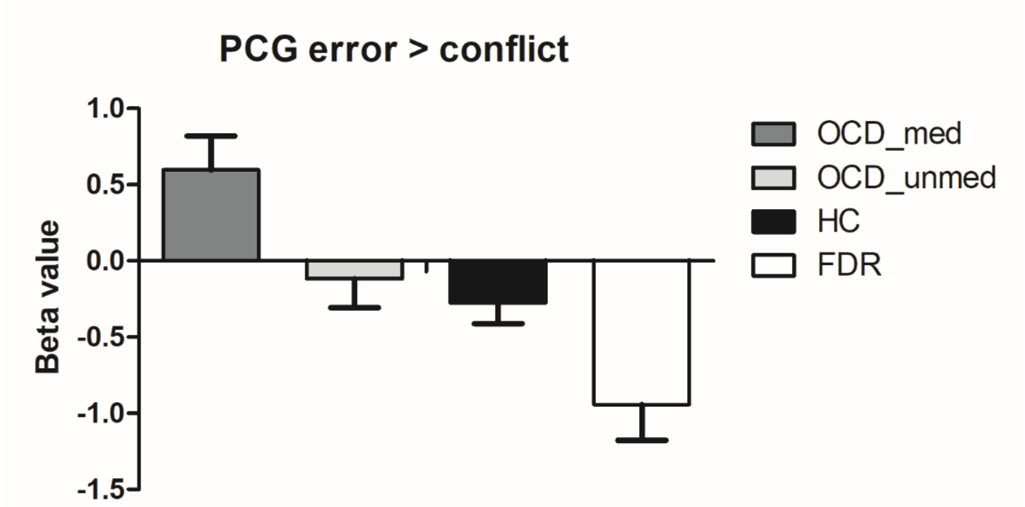

Figure S3. Error-related activity of the left postcentral gyrus (PCG) ± standard error in medicated OCD patients (OCD_med), unmedicated OCD patients (OCD_unmed), healthy control participants (HC) and unaffected first-degree relatives of OCD patients (FDR).

A similar pattern was observed for the postcentral gyrus. Error-related activation of the postcentral gyrus was stronger in medicated patients than in unmedicated patients, $t(82) = 2.16$, $p = .034$. Consequently, medicated patients showed stronger error-related postcentral gyrus activation than HC, $t(132) = 3.11$, $p = .002$, while the difference did not reach significance in unmedicated OCD patients, $t(146) = 0.78$, $p = .436$. FDR showed decreased activation of the postcentral gyrus compared to both patient groups, all t > -3.49, all $p < .001$, and to HC, $t(134) = -3.00$, $p = .003$.

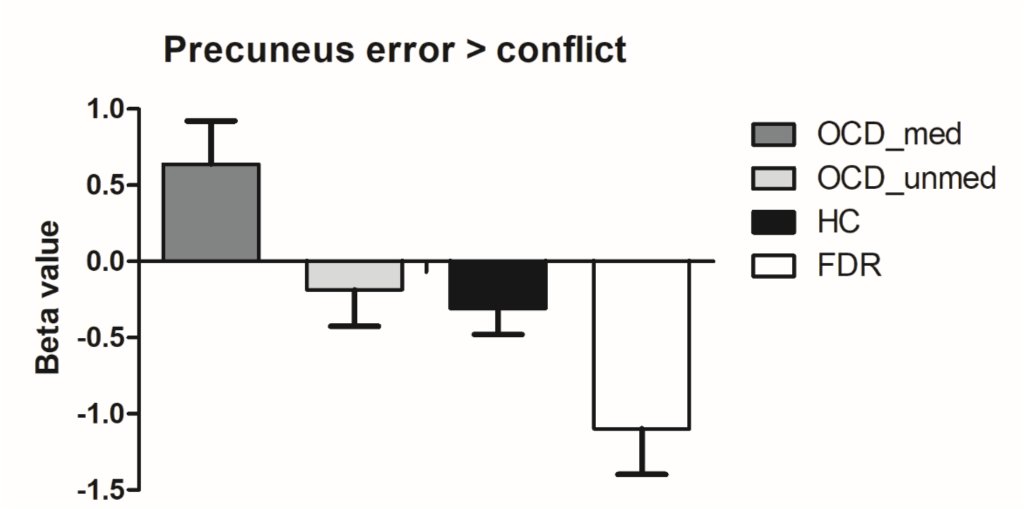

Figure S4. Error-related activity of the right precuneus ± standard error in medicated OCD patients (OCD_med), unmedicated OCD patients (OCD_unmed), healthy control participants (HC) and unaffected first-degree relatives of OCD patients (FDR).

The analysis for the precuneus further confirmed this pattern. Error-related activation of the precuneus was stronger in medicated patients than in unmedicated patients, $t(82) = 2.03$, $p = .046$. Consequently, medicated patients showed stronger error-related precuneus activation than HC, $t(132) = 2.80$, $p = .006$, while the difference did not reach significance in unmedicated OCD patients, $t(146) = 0.49$, $p = .628$. FDR showed decreased activation of the precuneus compared to both patient groups, all $t > -2.90$, all $p < .005$, and to HC, $t(134) = -2.91$, $p = .004$.

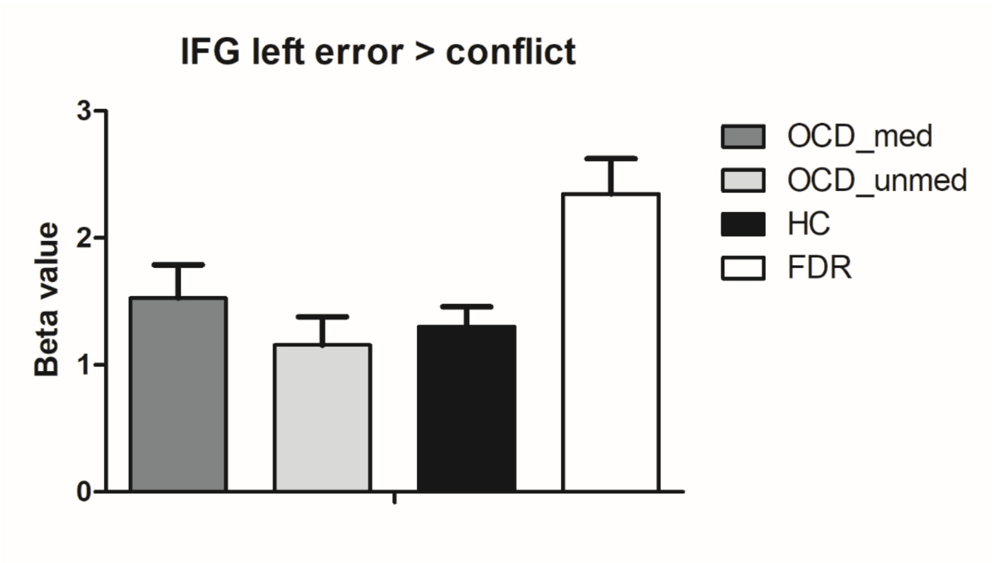

Figure S5. Error-related activity of the left inferior frontal gyrus (IFG) ± standard error in medicated OCD patients (OCD_med), unmedicated OCD patients (OCD_unmed), healthy control participants (HC) and unaffected first-degree relatives of OCD patients (FDR).

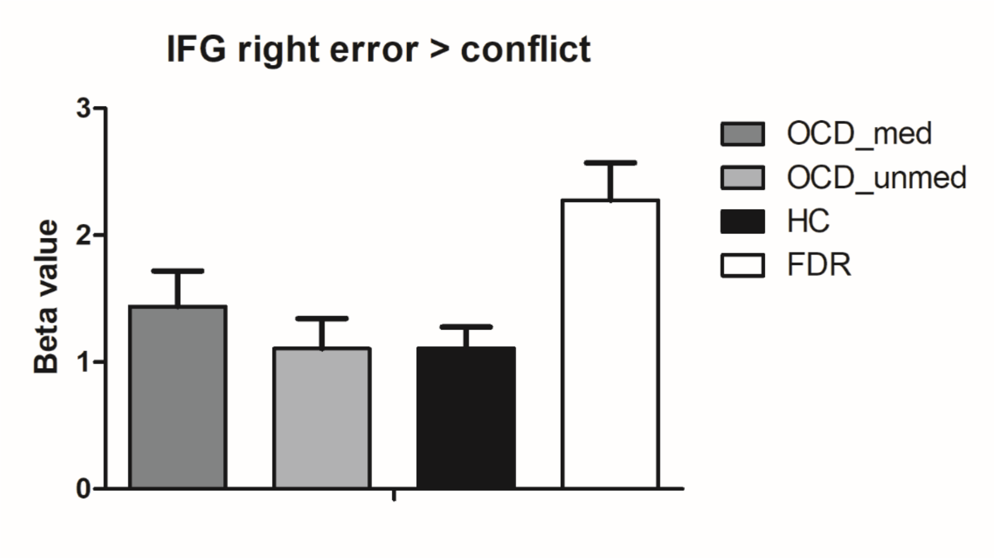

Figure S6. Error-related activity of the right inferior frontal gyrus (IFG) ± standard error in medicated OCD patients (OCD_med), unmedicated OCD patients (OCD_unmed), healthy control participants (HC) and unaffected first-degree relatives of OCD patients (FDR).

For the left and right IFG error-related activation was significantly higher in FDR than HC and unmedicated OCD patients (all $t > 2.18$, all $p < .032$), while no difference was observed in comparison to medicated OCD patients (all $t < 1.22$, all $p > .226$).

These results stand in contrast to previous studies and meta-analyses that showed no effect of medication on error-related activation of the MCC/SMA (5-7). Therefore, it appears unlikely, that the pattern observed in the present data is directly caused by physiological effects of the psychoactive medication. Importantly, patients were recruited from the outpatient clinic of the Humboldt University, where they were on the waiting list for cognitive-behavioral therapy. Thus, the patients in the medicated sample sought psychotherapy in addition to medication treatment. It appears plausible, that these patients were more severely ill and might have exhibited higher OCI-R and Y-BOCS scores before the start of medication treatment. As they have already experienced a slight, but unsatisfactory symptom reduction they do not significantly differ in symptom scores from unmedicated patients at the time of the study. This is supported by the numerically higher OCD and depression symptoms in the medicated group. Additionally, age of onset was numerically lower and illness duration numerically higher in medicated OCD patients. In line with this, recent analyses from the ENIGMA-OCD consortium demonstrated that structural brain volume alterations are selectively present in medicated OCD patients (8-10). Interestingly, in these mega-analyses medicated OCD patients also exhibited slightly higher OCD symptoms. Thus, the authors propose that these structural differences may partly be driven by pre-medication differences in illness severity. However, longitudinal designs are needed in order to assess the effects of medication on error-related brain activity. Taken together, the medication effect in the current sample further illustrates, that error-related hyperactivity of the MCC as measured with FMRI is less robust than ERN amplitude and thus less suited to serve as an endophenotype candidate/biomarker of OCD.

Supplemental References

1. Dutilh G, van Ravenzwaaij D, Nieuwenhuis S, van der Maas HLJ, Forstmann BU, Wagenmakers E-J (2012): How to measure post-error slowing: A confound and a simple solution. *Journal of Mathematical Psychology*. 56:208-216.
2. Sambataro F, Murty VP, Callicott JH, Tan HY, Das S, Weinberger DR, et al. (2010): Age-related alterations in default mode network: impact on working memory performance. *Neurobiology of Aging*. 31:839-852.
3. Schreiber M, Pietschmann M, Kathmann N, Endrass T (2011): ERP correlates of performance monitoring in elderly. *Brain and Cognition*. 76:131-139.
4. Larson MJ, Clayson PE, Keith CM, Hunt IJ, Hedges DW, Nielsen BL, et al. (2016): Cognitive control adjustments in healthy older and younger adults: Conflict adaptation, the error-related negativity (ERN), and evidence of generalized decline with age. *Biological Psychology*. 115:50-63.
5. Stern ER, Liu YN, Gehring WJ, Lister JJ, Yin G, Zhang J, et al. (2010): Chronic medication does not affect hyperactive error responses in obsessive-compulsive disorder. *Psychophysiology*. 47:913-920.
6. Riesel A (2019): The erring brain: Error-related negativity as an endophenotype for OCD-A review and meta-analysis. *Psychophysiology*. 56:e13348.
7. Norman LJ, Taylor SF, Liu Y, Radua J, Chye Y, De Wit SJ, et al. (2019): Error Processing and Inhibitory Control in Obsessive-Compulsive Disorder: A Meta-analysis Using Statistical Parametric Maps. *Biological Psychiatry*. 85:713-725.
8. Boedhoe PSW, Schmaal L, Abe Y, Alonso P, Ameis SH, Anticevic A, et al. (2018): Cortical Abnormalities Associated With Pediatric and Adult Obsessive-Compulsive Disorder: Findings From the ENIGMA Obsessive-Compulsive Disorder Working Group. *American Journal of Psychiatry*. 175:453-462.
9. Boedhoe PSW, Schmaal L, Abe Y, Ameis SH, Arnold PD, Batistuzzo MC, et al. (2017): Distinct Subcortical Volume Alterations in Pediatric and Adult OCD: A Worldwide Meta- and Mega-Analysis. *American Journal of Psychiatry*. 174:60-69.
10. Bruin WB, Taylor L, Thomas RM, Shock JP, Zhutovsky P, Abe Y, et al. (2020): Structural neuroimaging biomarkers for obsessive-compulsive disorder in the ENIGMA-OCD consortium: medication matters. *Translational Psychiatry*. 10:342.
